# Blood transfusion during cardiac surgery: massive transfusion coagulopathy

**DOI:** 10.1186/1749-8090-8-S1-P110

**Published:** 2013-09-11

**Authors:** A Trlaja, D Glavaš, C Bulat, M Carev, N Ipavec, B Pauković-Sekulić, I Bradarić, M Deković, N Karanović

**Affiliations:** 1 Department of Transfusion, University Hospital Center Split, Split, Croatia; 2 Department of Internal Medicine, University Hospital Center Split, Split, Croatia; 3 Department of Cardiothoracic Surgery, University Hospital Center Split, Split, Croatia; 4 Department of Anestesiology, Resuscitation and Intensive Care, University Hospital Center Split, Split, Croatia; 5 Haematology Laboratory, University Hospital Split, Split, Croatia

## Background

Massive transfusion is usually defined as transfusion of more than 10 units of packed RBCs within 24h. Trigger for red cell transfusion in cardiac surgical patients is HGB 100 g/L or HCT 0.30 L/L. We implemented control resuscitation with preemptive use of platelets and plasma in transfusion (1:1:1).

## Methods

Total of 406 patients (f/m N=103/303) were studied in the ICU in 2011; 21 (5.17%) males were observed,. HGB in OR prior to surgery was: 127.43 g/L(SD±19.4; range 85-165; median 129g/L). Patients were divided into two groups, according to the quantity of RBCs received. Group 1 (N=15) received 5-9 units RBCs; group 2 (N=6) received ≥10 units RBCs. EuroSCORE II was used for operation risk calculation. Patients abode 6.9 days (±7.97) in ICU. LVEF was 61.1±11.06%. The consumption of RBCs, platelet concentrates (PCs), and fresh frozen plasma (FFP) within 24 h was calculated. In all these cases CBC, PT, aPTT and INR were used to evaluate perioperative bleeding. Patients were monitored prior to surgery and for the first 24 h of transfusion of blood components for creatinine, pH, Ca++, troponin I. Data are presented as median and IQR; mean and standard deviation, P < 0.05 was considered statistically significant.

## Results

GROUP 1: (before/after) blood transfusion: PLT 171.47x109/L (±56.69) / PLT 139.53x109/L (±44.86); P=0.02. PV 0.85(0.62-0.97) / 0.94(0.31-1.113); aPTT 30 (26-36) / 30 (26-120); aPTT R 1.04(0,91-1.18) / 1.05 (0.91-1.27). GROUP 2:PLT 156.67x109 L (±76.26) / PLT 119.67x109 (±62.44);P=0.04. PV 0.78(53-1.09) / 0.94(0.34-1.00); aPTT 32(25-49) / 35.5 (27-50); aPTT R 1.10 (0.86-1.70) / 1.23(0.93-1.71). Patients who were massively transfused received: RBC 10.5(SD±3.93; r:10-20) units, FFP 11(SD±5.42; r:5-21) units, PCs 10 (SD±5.11; r:7-20) units.

## Conclusion

With this protocol we prevent bleeding following massive transfusion that can occure due to hypothermia, dilutional coagulopathy, platelet dysfunction, fibrinolysis, and hypofibrinogenemia.

